# Silica-coated magnetite nanoparticles core-shell spheres (Fe_3_O_4_@SiO_2_) for natural organic matter removal

**DOI:** 10.1186/s40201-016-0262-y

**Published:** 2016-11-25

**Authors:** Elahe Karimi Pasandideh, Babak Kakavandi, Simin Nasseri, Amir Hossein Mahvi, Ramin Nabizadeh, Ali Esrafili, Roshanak Rezaei Kalantary

**Affiliations:** 1 Department of Environmental Health Engineering, School of Public Health, Tehran University of Medical Sciences, Tehran, Iran; 2 Department of Environmental Health Engineering, Ahvaz Jundishapur University of Medical Sciences, Ahvaz, Iran; 3 Student Research Committee Ahvaz Jundishapur University of Medical Sciences, Ahvaz, Iran; 4 Research Center for Environmental Health Technology (RCEHT), Iran University of Medical Sciences, Tehran, Iran; 5 Department of Environmental Health Engineering, School of Public Health, Iran University of Medical Sciences, Tehran, Iran

**Keywords:** Humic acid, Hybrid adsorbent, Magnetic separation, Adsorption, Silica-magnetite

## Abstract

**Background:**

In this work, the magnetite (Fe_3_O_4_) nanoparticles (MNPs) and silica-coated magnetite nanoparticles (SMNPs) were synthesized as adsorbents for removing humic acid (HA) from water resources.

**Methods:**

The adsorption processes were performed in batch experiments with which the influence of pH, reaction time, adsorbent dosage, initial concentrations of HA and temperature were investigated. Specific techniques were applied to characterize the features of both adsorbents (i. e. TECHNIQUES) (SEM, XRD, TEM, BET, EDX and VSM).

**Results:**

The maximum saturation magnetization for SMNPs was 30.2 emu/g, which made its separation from the solution by a magnetic field to be easier and faster. The HA adsorption process onto the both adsorbents were best described by the Freundlich isotherm and pseudo-second-order kinetic models. Highest adsorption efficiency of HA by MNPs an d SMNPs occurred at acidic conditions (pH ≈ 3). The mechanisms of adsorption process involved with a physisorption process such as (i. e. hydrogen bonding and electrostatic interaction). The predicted maximum monolayer adsorption capacities obtained by Langmuir isotherm model for MNPs and SMNPs were 96.15 and 196.07 mg/g, respectively.

**Conclusion:**

Higher amount of HA adsorption onto the surfaces of SMNPs than MNPs surfaces was observed, reflecting that silica impregnated on MNPs enhances the efficiency of the adsorbent in removing HA.

**Electronic supplementary material:**

The online version of this article (doi:10.1186/s40201-016-0262-y) contains supplementary material, which is available to authorized users.

## Background

In general, the amount of natural organic matter (NOM) in drinking water resources is an important issue. In fact, it is best described as a complex mixture of organic compounds, particularly included with humic and fulvic acids; and also, the other organic compounds, including proteins, lipids, carbohydrates, carboxylic acids, amino acids, and hydrocarbons [1]. Although the disinfecting agents play an important role in keeping drinking water supplies safe, applying these agents could cause the formation of harmful disinfection by-products (DBPs) resulted from their reaction with NOM. From the qualitative point of view, the presence of NOM in the water could contribute to the formation of odor, poor color, and taste [2]. In addition, NOM, by forming complex with heavy metals, increases the solubility of these metals in the water; besides, reproduction of bacteria at the presence of NOM is facilitated which could jeopardize the health of people [3]. Furthermore, NOM has been reported to be able of stabilizing the dispersion of some potentially hazardous nanoparticles in the solutions which could pose potential threat to human health being and ecosystem [4].

During the last decades, researchers have strived to remove NOM from the water resources. In this regard, conventional treatment processes, including adsorption with different types of the adsorbents [5], coagulation [6], ion exchange [7], advanced oxidation [8], biodegradation [9] and membrane filtration [10] have been applied. Based on a conducted review focusing on this issue, one of the most applied methods for removing NOM is the adsorption, due to the following characteristics of this process: ease of application, simplicity and feasibility, and being reluctant against the contaminants as well as toxic substances [11]. Wide range of the adsorbents have been investigated with the effort to remove humic acid (HA) from water, including granular ferric hydroxide [12], magnetic chitosan nanoparticle [13], activated carbon, and newly developed nanoparticles (i. e. carbon nanotubes and chitosan/zeolite composites) [14, 15].

Over the last decade, nano-sized magnetic particles have been investigated comprehensively and increasingly developed. In fact, magnetic nanoparticles are a particular sort of nanoparticles which can be easily separated by means of an external magnet [16, 17]. In addition, these nanoparticles have enormous specific surface area, large quantity of the reactive surface sites, low toxicity, and excellent magnetic features [18]. Considering mentioned advantages of nano-sized magnetic particles, large adsorption efficiency, significant amount of pollutants adsorption, and quick separation of the adsorbent from the aqueous media through application of an external magnetic field can be expected from applying this method [17, 19, 20].

However, the most regular developed and applied magnetic adsorbents are based on iron oxides; in other words, iron oxides particles are susceptible to leach out under acidic conditions [21]. Moreover, the agglomeration of magnetic nanoparticles is another major concern in applying this method [22]. On one hand, coating the magnetic particles with biocompatible, high solubility in water and nontoxic materials could be an effective way to overcome aforementioned problems. On the other hand, some of the functional groups on the coating layer are firmly attached onto to the magnetic nanoparticles and vulnerable to acid treatment [18]. Several coating materials, including polymer, Au, and silica have been developed to modify the magnetic nanoparticles [17]. Among these materials, silica is the most ideal coating medium for the magnetic materials; in fact, this material is chemically inert which prevent it from affecting the redox reaction at the core. By suitable coating, the magnetic dipole-dipole attractions among nanoparticles might be covered which could result in minimizing or preventing aggregation [23].

The present study is aimed to synthesize MNPs with silica based coating layer (SMNPs) using chemical co-precipitation method. The structural, physical and chemical properties of both adsorbents were analyzed; and then, their efficiency in HA removal from aqueous solution was studied. The influencing variables on the adsorption were optimized and the isotherms, kinetics and thermodynamic studies of HA adsorption were carried out in detail. Furthermore, the adsorbents reusability and stability were also examined in a five consecutive cycles process.

## Methods

### Chemicals

Humic acid (residue on ignition about 20%), FeCl_3_.6H_2_O (96% *w/w*), FeCl_2_.4H_2_O, tetraethoxysilane (TEOS) (Si (OC_2_H_5_)_4_), and ammonia solution (NH^−^_3_.H_2_O) were obtained from Merck Co., Darmstadt, Germany. To prepare and wash the materials, we used absolute ethanol (96%). All the solutions used in this study were prepared using deionized DI-water. To adjust pH, HCl and NaOH solutions (0.1–1.0 M) were applied. The HA concentration was determined using a UV-Visible Spectrophotometer (CECIL-7100, UK). Furthermore, the residues of iron in the solution were measured by means of atomic absorption spectrophotometry (AAS, Shimadzu AA 6800).

### Synthesis of MNPs

MNPs was prepared based on the chemical co-precipitation method. According to this method, first, DI-water together with nitrogen gas was added during 30 min to not only to bring about adequate agitation, but also to hamper the ferrous ions oxidation. Afterwards, 4 g FeCl_3_.6H_2_O and 2 g FeCl_2_.4H_2_O were dissolved in 200 mL DI-water under nitrogen atmosphere with severely stirring at 80 °C. After that the solution were mixed for 30 min, then 20 mL NH ^−^_3_.H_2_O (28%) was poured drop wise into the solution until the pH raised to about 10 and the stirring continued for 45 min. When ammonium was being added to the solution, it was led to changing of solution color from the brown to dark brown and then became black. After the temperature of the synthesized adsorbent lowered, adsorbent was repeatedly washed using ethanol until reaching pH to the neutral state and which followed by drying in an oven at 105 °C for 4 h.

### Synthesis of SMNPs

Based on a modified co-precipitation technique, we synthesized SMNPs composite [11]. A solution of MNPs with 0.125 M (80 mL) was prepared via dispersing the MNPs in absolute ethanol at 40 °C. When the suspension was formed, it was mixed with 4 mL of 21% ammonia, 7.50 mL DI-water and 0.56 mL of TEOS for 2 h. After this, the suspension was sonicated using an ultra-sonic instrument (Elma, D-78224-P750W) for 1 h. The composite was magnetically separated from the suspension using a magnetic field and then dispersed in 30 mL of ethanol. For improving the Si–O–Fe bonding, the solution was kept in a 60 °C water bath for 6 h. Then, it was washed several times by alcohol until its pH became neutral; and also, it was gathered using an external magnet, vacuum-dried at 70 °C for 12 h, and ultimately kept in an air tight container.

### Physicochemical properties of the adsorbents

X-ray diffraction (XRD) (Quantachrome, 2000, NOVA) was applied to specify their crystallinity and degree of purity in both MNPs and SMNPs adsorbents. The Debye-Scherrer’s formula [24] was employed to determine the average diameter (D) of the crystallites of the MNPs particles as:

1 $$ \mathrm{D}=\frac{\mathrm{K}\uplambda}{\upbeta\ \mathrm{C}\mathrm{o}\mathrm{s}\ \uptheta} $$

Where, K is the Scherrer’s constant of the order of 0.89, λ is the wavelength of X-ray of Cu Kα radiation, β is the full width of half maximum of a diffraction peak, and θ is the diffraction angle.

SEM (PHILIPS, XL-30) was applied to determine the morphological surface of the adsorbents and incorporation state of MNPs onto the surface of silica. Transmission electron microscopy (TEM) images were taken on PHILIPS, EM with high-resolution at 100 kV to find the shape and size of the fabricated nanoparticles. The elemental analysis of the adsorbents was determined by means of energy dispersive X-ray (EDX, PHILIPS, XL-30) approach. In addition, for measuring the surface area, pores volume and average diameter of the synthesized adsorbents, the BET analysis (Quantachrome, 2000, NOVA) was used. Furthermore, a vibrating sample magnetometer (VSM) (7400, Lakeshare, USA) was employed to evaluate the magnetic features of MNPs and SMNPs.

### Batch experiments set-up and procedure

**Scheme 1:**
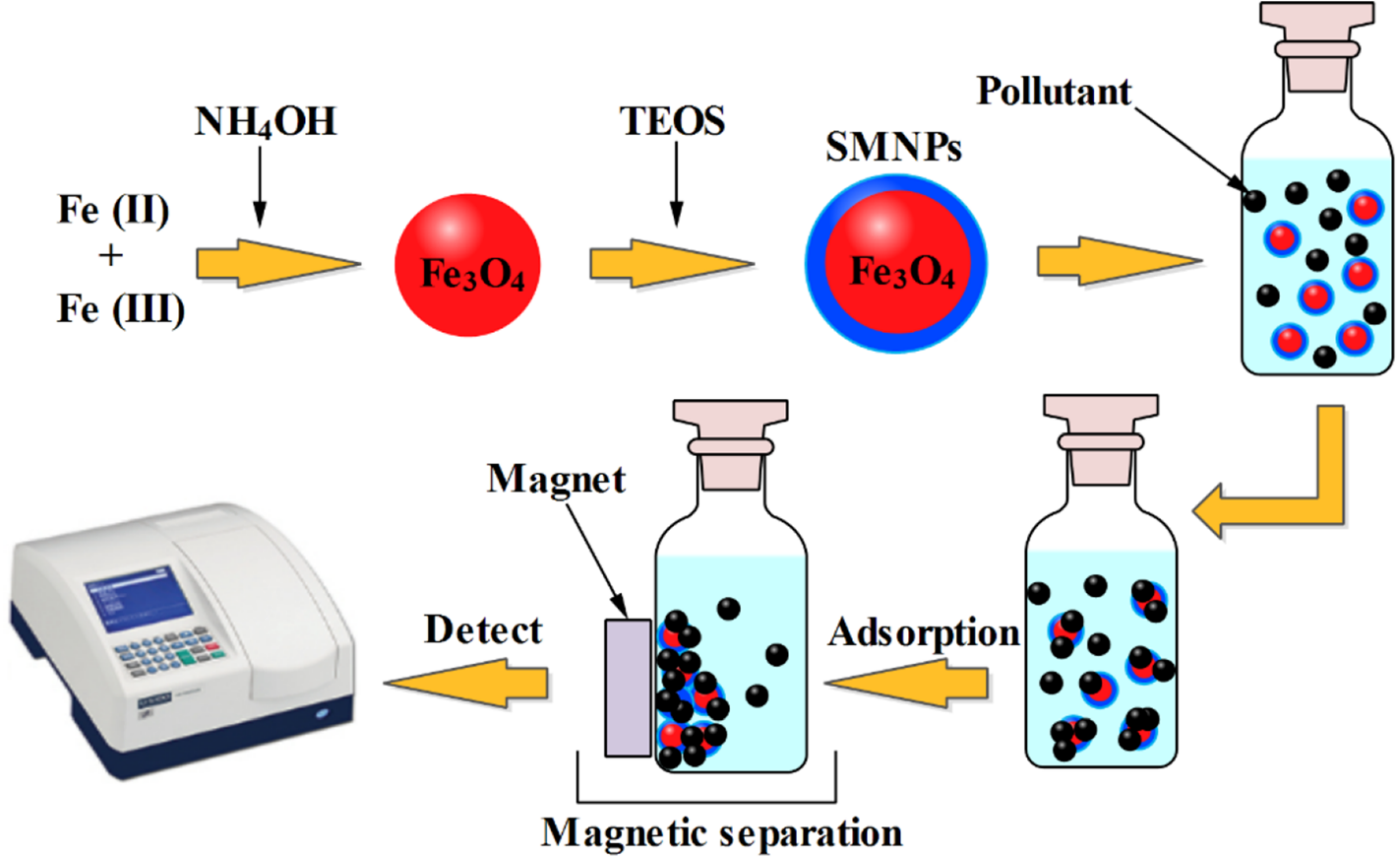
The schematic diagram of the synthesis of adsorbents and HA adsorption process on the SMNPs

The effect of the influencing variables, including pH, contact time, adsorbent dosage, initial concentrations of HA, and temperature on the adsorptive removal of HA was studied through batch experiments. To prepare HA stock solution (100 mg/L) a specific quantity of HA was dissolved in DI-water. It is worth to mention that the HA stock solution was diluted using DI-water to prepare HA solution with different concentrations. The experiments regarding the adsorption of HA onto the MNPs and SMNPs were carried out using 100 mL Erlenmeyer flasks containing 50 mL of HA solutions. Then, the flasks were vigorously shaken using a rotary shaker at 220 rpm and 20 ± 2 °C to meet the required adsorption equilibrium conditions. It should be mentioned that the solutions were taken at specified time intervals to separate the adsorbents from the solutions for less than 1 min via an external magnetic field. The residual HA concentration into the samples were detected by means of a UV-visible spectrophotometer at its maximum absorbance wavelength for HA (254 nm). Each of all the experiments was carried out for three times; and then, mean and standard deviation (SD) of the values were calculated and used to obtain the final results. The adsorption capacity (*q*_*e*_) and the adsorptive removal of HA were calculated using the equations below:

2 $$ {\mathrm{q}}_{\mathrm{e}}=\frac{\left({\mathrm{C}}_{\mathrm{o}}-{\mathrm{C}}_{\mathrm{e}}\right)}{\mathrm{W}} $$

3 $$ \mathrm{Adsorption}\left(\%\right)=\left(\frac{{\mathrm{C}}_{\mathrm{o}}-{\mathrm{C}}_{\mathrm{e}}}{{\mathrm{C}}_{\mathrm{o}}}\right)\times 100 $$

where, q_e_ (mg/g) is adsorbed amount of HA on the adsorbent per unit, *C*_*o*_ and *C*_*e*_ (mg/L) are the initial and residual HA concentration, respectively, and W (g/L) is defined as the amount of dry mass of the adsorbent per solution volume. The schematic diagram of different steps of the synthesis of MNPs and SMNPs as well as HA adsorption process has been illustrated in Scheme 1.

### Optimization of the adsorption process

To optimize the influencing variables on the HA adsorption, the influence of solution pH in the range of 3 to 11 was initially studied at 100 min contact time and room temperature. Afterwards, the influence of reaction time and the adsorption kinetics were tested on at initial HA concentrations of 10 mg/L for 4 h. Various dosages of MNPs and SMNPs in the range of 0.25 to 0.75 g/L were applied to be evaluated. Afterwards, various concentrations of the adsorbents (0.25–0.75 mg/L) were employed to analyze the adsorption isotherms. Meanwhile, the study of adsorption thermodynamics was developed at different temperatures (20–50 °C).

### Reusability and stability experiments

The reusability and regeneration of the applied adsorbents were studied using methanol (99.9%), DI-water and HCl extract the adsorbed HA. The adsorbents reusability was also carried out in five adsorption-regeneration cycles. In this regard, 0.5 g of each adsorbent (MNPs and SMNPs) was added to 100 mL solution contained with 10 mg/L HA and shaken for 4 h at 25 ± 1 °C and pH 3.0. The adsorbents were magnetically collected, rinsed and then followed by drying at 110 °C. Afterwards, the samples of 0.10 g of MNPs and/or SMNPs loaded with HA were added to 100 ml of HCl as desorbing solutions and being shaken for 24 h at 200 rpm and 25 ± 1 °C. Then, the rate of HA desorption (%) was quantified through Eq. (4). Where C_e(des)_ and C_e(ads)_ are the residual HA concentrations in the solution after the desorption and adsorption, respectively. After the desorption, the regenerated adsorbents were dried in an oven at 100 °C for 60 min to be applied for the subsequent adsorption-regeneration cycle with the purpose of testing the MNPs and SMNPs reusability in removing HA. After ending every adsorption-desorption cycle, the adsorbents stability was determined through measurement of the iron concentration in the solution:

4 $$ \mathrm{Desorption}\left(\%\right)=\frac{{\mathrm{C}}_{\mathrm{e}\left(\mathrm{d}\mathrm{e}\mathrm{s}\right)}}{{\mathrm{C}}_{\mathrm{e}\left(\mathrm{ads}\right)}}\times 100 $$

## Results and discussion

### Chemical and textural features of MNPs and SMNPs

**Fig. 1:**
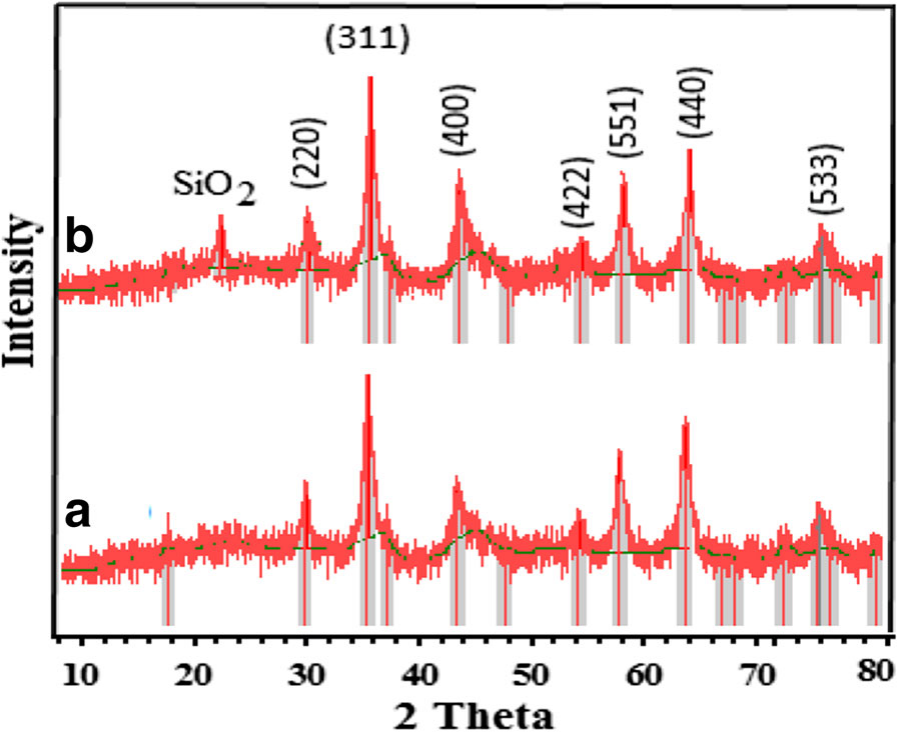
XRD spectrum of MNPs before (**a**) and after (**b**) silica coating

**Fig. 2:**
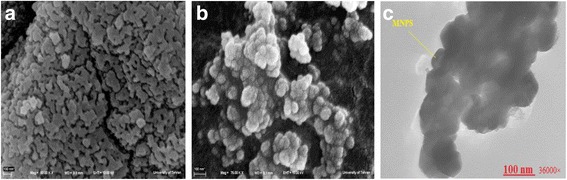
SEM images of MNPs before (**a**) and after (**b**) silica coating and TEM micrograph of SMNPs (**c**)

X-ray diffraction (XRD) analysis is a promising analytical tool for characterization of physical and chemical forms of the magnetic particles incorporated in the silica body. The diagrams of XRD of the MNPs and SMNPs were prepared in 2*θ* ranged from 10 to 80° (Fig. 1). The main peaks at 2*θ* equated 30.1, 35.4, 43.1, 53.4 56.9, 62.5 and 74.9° for MNPs, which were marked respectively with (220), (311), (400), (422), (511), (440) and (533) indices based on the plane of a cubic spinel structure of the MNPs (JCPDS card no. 19–0629) [11]. In addition, similar peaks were seen in the XRD diagrams of synthesized SMNPs composite, confirming that the cubic phase of MNPs is still kept after incorporating with the mesoporous silica. This observation is consistent with the obtained findings of the previously performed research in this regard [25, 26]. In addition, a weak peak (2*θ* =22°) was also seen in the diagram of SMNPs composite, belonging to amorphous silica (JCPDS No. 29–0085) [27]. Considering this, it can be implied that SiO_2_ particles were present in the structure of SMNPs composite. The average particle size of the MNPs based on the Scherrer’s equation was obtained ranging from 80 to 108.5 nm. Figure 2 shows the SEM images of MNPs and SMNPs at 10.0 keV. From Fig. 2a, it can be seen that the external surface MNPs is almost flat and has some cavities; and also, the agglomerate of MNPs was observed. It was found that the average size of MNPs is 100 nm, confirming that the MNPs of Fe_3_O_4_ have been successfully synthesized at nano-size scales. However, in comparison with MNPs, SMNPs (see Fig. 2b) had an irregular as well as heterogeneous surface; also, it is noteworthy that most of the SMNPs are spheroid. Furthermore, it indicates that the MNPs (white color) have a non-uniform distribution way. The mean of SMNPs particles size was obtained in the range of 30 to 130 nm. TEM micrograph (Fig. 2c) shows that the Fe_3_O_4_ particles were almost well dispersed within silica surface with the average side length of 100 nm. It also reveals a spherical structure for MNPs which is in line with the findings of XRD analysis. It also clearly shows silica coating layer on the surface of MNPs.

**Fig. 3:**
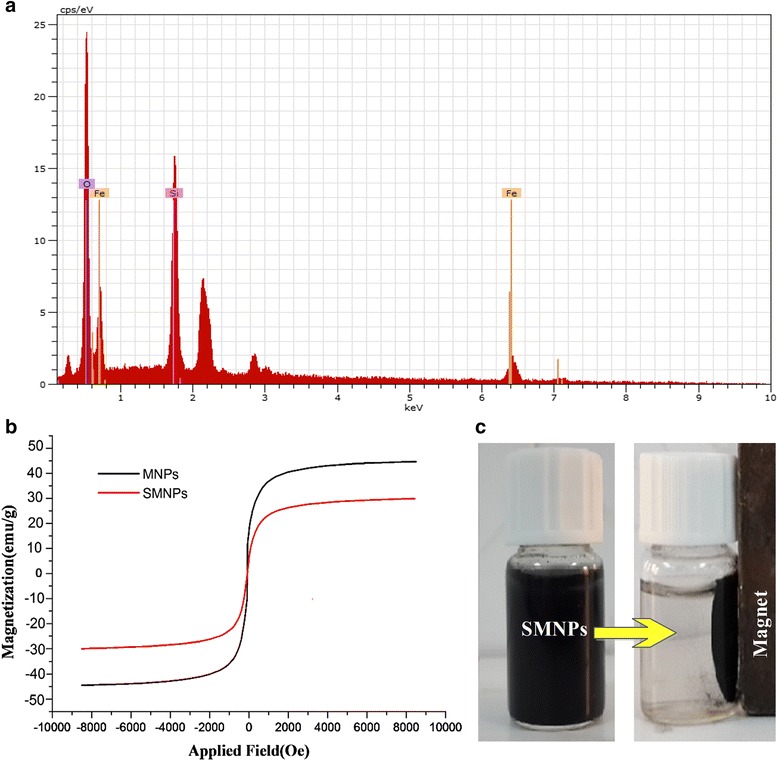
**a** EDX peaks for SMNPs, **b** the room temperature hysteresis loops of MNPs and SMNPs and **c** magnetically separation of SMNPs from aqueous solution by using external magnet field

EDX analysis of MNP_s_ (Fig. 3a) represents some peaks indication some elements such as carbon (C), oxygen (O), and iron (Fe) in the body of synthesized composite was assured. In other words, it illustrates that the SMNPs composite is composed of 18.02% silica, 25.88% oxygen and 56.1% iron. Some peaks related to the iron in EDS analysis proved the presence of iron in the body of SMNPs composite. This observation is also consistent with the result of X-ray diffraction analysis. Hence, it can be concluded that 32% of the surface of MNPs was covered with silica. Figure 3b represents the diagram of VSM magnetization of the synthesized adsorbents at 25 °C in the cycling magnetic field of ±10 kOe. The highest magnetization saturation for MNPs and SMNPs was respectively 44.8 and 30.2 emu/g. Considering that neither coercivity nor remanence was observed, it can be implied that the two adsorbents are super-paramagnetic. The magnetization value for SMNPs composite was less than its corresponding rate in the synthesized MNPs. As a matter of fact, this results can be derived from the existence of non-magnetic silica onto the body of nanoparticles. Nevertheless, the saturation magnetization of SMNPs was lower than pure MNPs, magnetic separation is still rapid and finished within less than 1 min completely in the presence of an external magnet. Based on these results, as-synthesized adsorbent (i. e. SMNPs) have good magnetic responsibility to a magnetic field (Fig. 3c). Therefore, it can be implied that this adsorbent can be used as a magnetic adsorbent to efficiently treat contaminated-aqueous environments and prevent a secondary pollution.

The specific surface area, the volume and mean size of the pores of both adsorbents were determined using BET analysis; and, the findings are presented in Table 1. The maximum surface area of MNPs and SMNPs were 98.7 and 272.5 m^2^/g, respectively. In other words, the SMNPs may provide a higher adsorption capacity in removing the contaminants, compared to MNPs’ capacity. The increase of the specific surface area for SMNPs can be due to the presence of silica in SMNPs textural. In addition, the mean pore size of MNPs and SMNPs were 3.1 and 3.7 nm, respectively. Based on this result and the IUPAC category, both adsorbents prepared in this study can be categorized into mesopores groups [28]. The lesser amount of the surface area, mean size and volume of pores of MNPs in comparison with SMNPs, could be due to the agglomeration of MNPs.

**Table 1:** Nitrogen sorption characteristics of the MNPs and SMNPs adsorbents used

Adsorbent	S_BET_, m^2^/g	Langmuir specific surface area, m^2^/g	Pore volume, cc/g	Average pore diameter, nm
MNPs	98.7	118.3	2.58	3.1
SMNPs	272.5	296.4	4.7	3.7

### Effect of pH

**Fig. 4:**
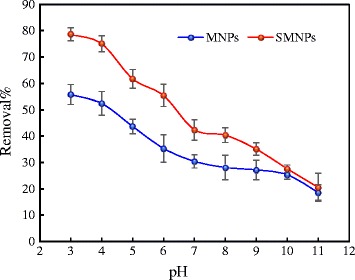
Effect of pH on the adsorption of HA by MNPs and SMNPs adsorbents (conditions: C_0_ = 10 mg/L, W = 0.5 g/L, t = 100 min, agitation speed = 200 rpm and T = 20 ± 2 °C)

Considering the effect of pH on solution chemistry, the surface charge of the adsorbent, and surface functional groups, this variable indeed has an outstanding pattern in the adsorption process [29]. As shown in Fig. 4, when pH of the solution increases from 3 to 11, the removal efficiency of HA by MNPs and SMNPs decrease from 55.8 and 78.6% to 18.6 and 20.5%, respectively. It proves that HA adsorption onto the MNPs and SMNPs surfaces is high in acidic pH conditions, attributing to the changes of surface features of the adsorbent and adsorbate. In fact, the attraction forces between HA molecules with negative charges and the adsorbent surfaces with positive charges caused high adsorption efficiency at pH 3. In a similar research performed by Wang et al. [30] it was found that the HA molecules is negatively charged between pH 3 and 10 in aqueous solution, due to the deprotonation of carboxylic and phenolic groups. In addition, Erhayem et al. [1] reported that the concentration of deprotonated carboxylic and phenolic species of NOM increase by enhancing the solution pH. Therefore, the removal efficiency can be enhanced through electrostatic interaction between HA molecules and the positively charged surfaces of MNPs and SMNPs. Moreover, at acidic conditions, the competition for the surface sites of the adsorbents between the molecules of HA and the large number of H^+^ cations does not exist in the solution, which result in comparatively high adsorption rate [31]. At basic pH conditions, however, the surfaces of the adsorbents have negative charges, and the electrostatic repulsion between HA molecules and the adsorbents could hamper HA molecules from reaching to the surface of the adsorbents; in fact, it might decline the adsorption efficiency of HA onto the surfaces adsorbent [30]. When pH increases, the HA molecules become less coiled and also compact, due to a greater charge repulsion, which could contribute to the decrease in the adsorption capacity [32]. Furthermore, similar results have been reported in the previously conducted research in terms of this issue [33, 34].

### Models and mechanism of HA adsorption

#### Adsorption kinetics

The adsorption kinetic is a crucial subject in batch studies than not only understand the sorption mechanisms, but also find the contribution of each experimental variables on the adsorption process [35]. In addition, the results of batch adsorption kinetics play a significant role in the design of industrial adsorption columns [20]. In the present research, the dominated mechanism in the process of HA adsorption on the MNPs and SMNPs was studied by means of linear kinetic equations. The experimental data analysis was carried out using some selected widely-used kinetic models, including pseudo first-order, pseudo second-order and intraparticle diffusion. The kinetic experiments of HA adsorption were carried out in a period of 4 h and at 20 ± 1 °C. It is noteworthy that further details of these models are shown in Additional file 1.

The relationship between the experimental and theoretical data was investigated using correlation coefficients (R^2^), R_adj_^2^ and error analysis. In addition, the applied R^2^, R_adj_^2^ and Ferror (%) are respectively based on the following Eqs. (5)-(7),:

5 $$ {\mathrm{R}}^2=\left(\frac{{{\displaystyle \sum_i^{\mathrm{n}}\left({q}_{e, \exp }-{\overset{-}{q}}_{e, \exp}\right)}}^2-{\displaystyle \sum_i^n{\left({q}_{e, \exp }-{q}_{e,t}\right)}^2}}{{\displaystyle \sum_i^{\mathrm{n}}\left({q}_{e, \exp }-{\overset{-}{q}}_{e, \exp}\right)}}\right)\times 100 $$

6 $$ {\mathrm{R}}_{\mathrm{adj}}^2=1-\left(1-{\mathrm{R}}^2\right)\ .\ \left(\frac{{\mathrm{n}}_{\mathrm{p}}-1}{{\mathrm{n}}_{\mathrm{p}}-\mathrm{p}}\right) $$

7 $$ {\mathrm{F}}_{\mathrm{error}}\ \left(\%\right)=100\times \sqrt{\left(\frac{1}{n_p-p}\right).{\displaystyle {\sum}_i^n{\left(\frac{q_{e, \exp }-{q}_{e,t}}{q_{e, \exp }}\right)}^2}} $$

where, q_e,t_ (mg/g) and q_e,exp_ (mg/g) represent the value of theoretically (i. e. by the fitted model) and experimentally measured q, respectively; n_p_ is the number of performed experiments and p is the number of parameters of the fitted model [22, 35].

**Fig. 5:**
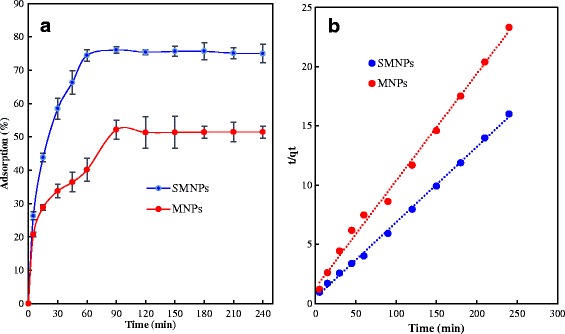
**a** Effect of contact time on adsorption of HA on MNPs and SMNPs (condition: pH = 3.0; agitation speed = 200 rpm; W = 0.5 g/L, C_0_ = 10 mg/L and T = 20 ± 2 °C) and **b** pseudo second-order adsorption kinetics of HA adsorption onto MNPs and SMNPs

The reaction time between the adsorbate and adsorbent is another critical parameter affecting the efficiency of the process of adsorption. The effect of reaction time on HA removal efficiency using both adsorbents was evaluated during a 0 to 240 min time period and at the optimized pH 3.0 (Fig. 5a). Based on the results, the HA adsorption rate on MNPs and SMNPs was initially fast which was followed by a slight decline until reached to the equilibrium state without any adsorption. The equilibrium point of HA adsorption onto both adsorbents was met at 90 min which was selected as an equilibrium time for the subsequent batch experiments. It should be mentioned that the adsorption equilibrium is the point at which the adsorbate concentration into the solution is in a dynamic balance with that of the interface [22]. Increasing HA removal rate by MNPs and SMNPs could be related to the abundance of vacant reactive sites on the surface of the adsorbents. It is observed in Fig. 5a, HA adsorption capacity by MNPs and SMNPs were almost constant after 90 min of operating this process. This phenomenon is principally derived from the fact that the number of accessible reactive sites for HA molecules declines when the contact time enhances [35, 36]. In other words, by passing the reaction time, the abundance of the adsorbate to the vacant reactive sites onto the adsorbent surfaces declines, leading to their prompt saturation of since the process reaches its equilibrium state [37].

Table 2 reports the obtained kinetic parameters of HA adsorption on MNPs and SMNPs. The correlation coefficients were found to be less than 0.94 and 0.84 for the pseudo first-order and intraparticle diffusion kinetic models, respectively; whereas, the corresponding amount calculated for the pseudo second-order kinetic model was 0.99. In fact, results showed the better fit of pseudo second-order with the data of HA adsorption with significant high coefficient of correlation (*R* ^*2*^) (>0.99), compared to the other kinetic models. In addition, the higher R^2^_adj_ and lower F_error_ (%) calculated for the pseudo second-order kinetic model, in comparison with their corresponding amount for the other two models, confirm this result. In other words, the pseudo second-order kinetic model better explains HA adsorption on MNPs and SMNPs adsorbents, than the other models. This model also depicts that the chemisorption was the rate-limiting step the adsorption process of HA onto the MNPs and SMNPs and there was no mass transfer reaction [21, 38]. Moreover, as shown in Table 2, it can be seen that the calculated *q*_e_ values of pseudo-second-order model are in line with the experimental *q*_e_ values; in other words, the adsorption process is best fitted to the pseudo second-order model, which is also shown in Fig. 5b. In previously conducted studies in this regard, the same model for the HA sorption on various adsorbents, like multi-walled carbon nanotubes [32], magnetic chitosan nanoparticle [13], and chitosan/zeolite composites [15] were reported.

**Table 2:** Parameters related to the kinetic models of HA adsorption on MNPs and SMNPs adsorbents

Kinetic models	Parameters	Adsorbent	
		MNPs	SMNPs
Pseudo first-order: *ln(q*_*e*_ *-q*_*t*_ *) = lnq*_*e*_ *-k*_*f*_ *t* *plot: ln(q*_*e*_ *-q*_*t*_ *) vs. t*	*q*_*e, Cal*_ *(mg/g)*	9.7	15.81
	*k*_*1*_ *(min* ^*−1*^ *)*	0.014	0.057
	h_0_ (mg/g. min)	0.135	0.9
	*R* ^*2*^	0.8417	0.9465
	*R*_*adj*_^*2*^	0.802	0.933
	*F*_*error*_ *(%)*	3.8	1.93
Pseudo second-order: *t/q*_*t*_ *= t/q*_*e*_ *+ 1/k*_*s*_ *q*_*e*_^*2*^ *plot: t/q*_*t*_ *vs. t*	*q*_*e,Cal*_ *(mg/g)*	11.1	15.72
	*k*_*2*_ *(g/mg) (min* ^*−1*^ *)*	0.006	0.01
	h_0_ (mg/g. min)	0.74	2.47
	*R* ^*2*^	0.995	0.9985
	*R*_*adj*_^*2*^	0.994	0.995
	*F*_*error*_ *(%)*	1.59	1.09
Intraparticle diffusion: *q*_*t*_ *= k*_*i*_ *t* ^*0.5*^ *plot: q*_*t*_ *vs. t* ^*0.5*^	*k*_*id*_ *(mg/gmin* ^*0.5*^ *)*	0.598	0.84
	*C*_*i*_ *(mg/g)*	2.724	4.86
	*R* ^*2*^	0.8406	0.7332
	Experimental q_e_ (mg/g)	10.5	15.22

As shown in Table 2, the h_0_ values obtained for SMNPs were significantly higher than the recorded values for MNPs. In other words, the kinetic of the adsorption of HA on SMNPs is faster than those obtained using MNPs. This issue can also be verified by comparing the kinetic rate constants (k_1_, k_2_ and k_id_) of the adsorbents with each other. In this regard, it can be implied that coating MNPs by silica promotes the macropores structure of MNPs, which contribute to higher amounts of the HA to be adsorbed by the SMNPs.

In addition, by using the intraparticle diffusion model, the influence of mass transfer resistance on the bond made between the adsorbate and the adsorbent can be evaluated [16]. In this study, to understand whether the intraparticle diffusion model is the main step in controlling the process of adsorption of HA onto MNPs and SMNPs adsorbents, the results for kinetic adsorption were fitted with the intraparticle diffusion models. In this regard, the model indicated that HA is probably transported from the solution to the adsorbents by intraparticle diffusion. As shown in Table 2, the values of correlation coefficients (*R* ^2^) for the intraparticle diffusion model are lower than those obtained from the pseudo second-order kinetic model; in other words, this model is not used. Furthermore, since the y-intercept (Ci) is not zero, it can be concluded that the intraparticle diffusion is part of the adsorption but not the only rate-controlling step in this process. It should be noted that other mechanisms (i. e. complexes or ion-exchange) affected controlling rate of the HA adsorption [38, 39].

#### Adsorption isotherms

The adsorption isotherms are known through specific constant values. In fact, the obtained constants indicate the surface features of the adsorbent and their tendency towards it. In this study, Langmuir, Freundlich and Temkin equilibrium isotherm models were used to express the adsorption behavior of HA onto the MNPs and SMNPs in order to describe the adsorption capacity. The theory of Langmuir and Freundlich isotherms expresses that the adsorption of a molecule on surfaces of the adsorbent follows the homogeneous and heterogeneous approaches, respectively. Moreover, the Temkin isotherm model proposes that several indirect interactions between adsorbent and adsorbate influence on the adsorption isotherms [38].

The adsorption isotherm experiments were conducted by using 1 to 50 mg/L HA and different adsorbent dosages (i. e. ranging from 0.25 to 0.75 g/L) under the optimized conditions at 25 ± 2 °C. Further details of these models are shown in Additional file 1.

The basic features of the Langmuir isotherm can be explained via a dimensionless separation factor R_L_ (i. e. R_L_ = 1/(1 + K_L_C_0_)). R_L_ values, in fact, indicate the type of isotherm. In other words, undesirable, desirable, irreversible, and liner adsorption occur when R_L_ > 1, 0 < R_L_ > 1, R_L_ = 0, and R_L_ = 1, respectively [35, 39].

In designing the adsorption systems, analysis of the equilibrium data is of high importance. Having a closer look to Table 3, the maximum adsorption capacity (q_o_) of HA on the MNPs and SMNPs decreased with adsorbent dose. Based on the results, it was found that increasing the adsorbent dosage can be attributed to the particle interactions (i. e. aggregation); in fact, it could result from high adsorbent concentrations which could led to the decreasing the amount of active surface area of the adsorbent which resulted in the reduction of adsorption capacity. This can also be explained by either the split in the flux or the concentration gradient between solute concentration in the HA concentration in the solution and the HA concentration in the adsorbent surface, resulting in that then amount of HA adsorbed onto unit weight of adsorbent gets reduced with increasing adsorbent dosage [15, 40]. In this regard, it can be speculated that the reason for this behavior has to do with the decrease of the ratio of adsorbate per mass unit of the adsorbent [41].

**Table 3:** Isotherms coefficients for the adsorption of HA onto MNPs and SMNPs at different adsorbent dosages (condition: pH = 3.0; agitation speed = 200 rpm; W = 0.25–0.75 g/L, C_0_ = 1–50 mg/L, t = 90 min and T = 20 ± 2 °C)

Adsorbent dose (g/L)	Langmuir				Freundlich			Temkin		
	Eq: C_e_/q_e_ = C_e_/q_0_ + 1/K_L_q_0_ plot: (C_e_/q_e_) *vs.* C_e_				Eq: lnq_e_ = lnK_F_ + n^−1^ lnC_e_ plot: ln q_e_ *vs.* ln C_e_			Eq: q_e_ = B_1_ ln K_T_ + B_1_ ln C_e_ plot: q_e_ *vs.* ln C_e_		
	q_m_ (mg/g)	K_L_ (L/mg)	r^2^	R_L_	K_F_ (mg/g(L/mg)^1/n^	n	r^2^	k_T_	q_m_	r^2^
MNPs										
0.25	96.15	0.024	0.9578	0.45–0.97	2.53	1.2	0.9952	0.85	11.36	0.8794
0.5	95.23	0.02	0.9738	0.5–0.98	2	1.13	0.9972	0.98	8.94	0.8633
0.75	62.9	0.04	0.9818	0.5–0.98	2.45	1.2	0.995	1.44	7.4	0.875
SMNPs										
0.25	196.07	0.041	0.9571	0.32–0.96	8.05	1.22	0.9982	1.55	22.8	0.8583
0.5	101.1	0.078	0.8523	0.2–0.93	7.36	1.33	0.996	2.84	12.6	0.8301
0.75	67.6	0.283	0.8971	0.06–0.78	12.7	1.66	0.9977	8.04	14.4	0.768

As shown in Table 3, the correlation coefficient (*R* ^*2*^) of Freundlich model was higher than obtained R^2^ for the Langmuir and Temkin models. Considering this result, Freundlich model is a better fit to the experimental data from the HA adsorption by MNPs and SMNPs than the other two models. It suggests that the heterogeneous functional sites have uniform distribution on the surface of both the adsorbents [42]. In other words, it shows that the adsorption of HA molecules onto non-energetically equivalent sites of the MNPs and SMNPs. In this regard, previously conducted research has also shown that the Freundlich isotherm model in comparison with the other models had the highest ability to fit experimental data of HA adsorption on different adsorbents [32, 43, 44]. Additional file 1: Figure S1 indicates the linear Langmuir, Freundlich and Temkin isotherms for HA adsorption onto the both adsorbents. The adsorption desirability was verified by taking the Freundlich exponent n into account, since its values for both the adsorbents were 0 < n < 1. Moreover, the values of 0 < R_L_ < 1 in the Langmuir model implies that HA is desirably adsorbed by the MNPs and SMNPs.

The maximum adsorption capacities (q_m_) of the MNPs and SMNPs were compared with the HA adsorption capacities of several studied adsorbents, which are shown in Table 4. Based on the Langmuir equilibrium model, the maximum amount of HA uptake per unit mass of MNPs was 96.15 mg/g; while, its corresponding amount for SMNPs was and 196.07 mg/g. Adsorption amount of HA on the SMNPs is significantly higher than that of MNPs, implying that silica on the surface of MNPs could enhance the adsorption of HA. It is worth mentioning that the MNPs and SMNPs poses a better adsorption capacity, compared to the capacity of other adsorbents applied in previous researches. In fact, a significant difference between the capacity of each adsorbent may be derived from their physical and chemical features like morphology, structure and reactive available sites. As shown in Table 4, the value of q_m_ for SMNPs is higher than that obtained for MNPs, by approximately 200%. This means that the silica had positive effect on HA removal via providing larger surface area, which is in line with the findings of BET analysis.

**Table 4:** Maximum adsorption capacity of some adsorbents for HA removal from aqueous media

Adsorbent	pH	Thermodynamic	Isotherm	Kinetic	q_m_ (mg/g)	Ref.
Magnetic chitosan nanoparticle (MCNP)	4.0	endothermic	Langmuir	Pseudo-second order	32.6	[13]
Chitosan/zeolite composites	4	endothermic	Langmuir	Pseudo-second order	74.1	[15]
Fe_3_O_4_@SiO_2_–PANI	2.0	endothermic	Langmuir	Pseudo-second order	36.36	[30]
MWCNTs	3.0	endothermic	Freundlich	Pseudo-second order	83.66	[32]
MWCNTs-COOH	3.0	endothermic	Freundlich	Pseudo-second order	50.26	[32]
Unburned carbon	3.0	endothermic	Freundlich	-	71.8	[43]
Fly ash	3.0	endothermic	Freundlich		10.7	[43]
Chitosan–ECH bea	6	-	Freundlich	Pseudo-first-order	44.84	[44]
Chitin	2.4	-	Langmuir	-	27.3	[48]
Chitosan	3.07		Langmuir	-	28.8	[48]
Montmorillonite-Cu(II)/Fe(III) oxides	3.1–6.1	-	Langmuir	-	98	[49]
Activated carbon (rice husk)	3.0	endothermic	Langmuir	-	45.4	[50]
Polyethylenimine functionalized magnetic mesoporous silica composite microspheres (MS-PEI)	5.5	-	Freundlich	Pseudo-second order	128.64	[51]
Alumina-pillared clays (Al-PILCs)	3.0	-	Langmuir	-	23.4	[52]
MNPs (Fe_3_O_4_)	3.0	endothermic	Freundlich	Pseudo-second order	96.15	This work
SMNPs (silica coated with Fe_3_O_4_)	3.0	endothermic	Freundlich	Pseudo-second order	196.07	This work

### Thermodynamics of the adsorption process

Thermodynamic studies of HA adsorption were investigated by applying 0.5 g/L dosage of the adsorbents, and 10 mg/L of HA at pH 3.0 and contact time of 90 min; also, the applied temperatures were 20, 30, 40 and 50 °C. In addition, change in free energy (ΔG°), enthalpy (ΔH°) and entropy (ΔS°), regarded as main factors, were calculated via given equations in Table 5. Furthermore, these parameters are discussed in details in Additional file 1.

**Table 5:** Thermodynamic parameters of HA adsorption on MNPs and SMNPs at different solution temperatures (conditions: pH = 3.0; agitation speed = 200 rpm; W = 0.5 g/L, C_0_ = 10 mg/L and t = 90 min)

Adsorbent	T(°C)	K_d_ (mL/g) [K_d_ = q_e_/C_e_]	ΔG° (KJ/mol) [ΔG° = −RT lnK_d_]	ΔH° (KJ/mol) $\ln {K}_d=\frac{\varDelta {S}^o}{R}-\frac{\varDelta {H}^o}{RT}$	ΔS° (J/mol) $\ln {K}_d=\frac{\varDelta {S}^o}{R}-\frac{\varDelta {H}^o}{RT}$
MNPs	20	0.492	−1.2	−6.626	−18.32
	30	0.484	−1.22		
	40	0.296	−0.77		
	50	0.279	−0.75		
SMNPs	20	1.54	−3.75	−2.31	−8.25
	30	1.5	−3.77		
	40	1.5	−3.9		
	50	1.48	−3.97		

Considering the thermodynamic parameters, their values at various temperatures are reported in Table 5. According to the obtained findings, the values of enthalpy (∆H°) for the adsorption of HA on the MNPs and SMNPs were −6.62 and −2.31 kJ/mol, respectively. In other words, the adsorption process was exothermic in nature. It should be noted that the magnitude of enthalpy (∆H°) indicates the either chemical or physical nature of adsorption. Normally, the obtained amounts of ∆H° were 2.1–20.9 kJ/mol for physical and 80–200 kJ/mol for chemical adsorption [13]. As shown in Table 5, the ∆H° values of HA adsorption onto both adsorbents were from 2.1 to 20.9 kJ/mol; in other words, the adsorption of HA onto MNPs and SMNPs involves a physisorption process (i. e. hydrogen bonding and electrostatic interaction). Similar results have been reported in previous research on the adsorption of HA onto the various substrate [13, 15]. However, the negative values obtained for the Gibbs’s free energy (ΔG°) demonstrate that the adsorption of HA on both adsorbents occurred spontaneously [38, 45]. The negative values also indicate that the reaction rate is decreasing and has an indirect relationship with the temperature. The negative values of ΔS° revealed a decrease in randomness at the solid-liquid interface in the adsorption system within the adsorption of HA onto the adsorbents [20, 41].

### Desorption and reuses

The adsorbent regeneration and restoration are two critical factors in the process of adsorbent applicability. As can be seen from Table 6, the reusability of both adsorbents did not change considerably after passing the five consecutive adsorption cycles. So that, HA sorption onto the MNPs and SMNPs decreased from 71.78 and 98.85% to 64.3 and 89.96%, respectively, after five cycles. This, in fact, proves that both adsorbents can be recycled and reused for at least five successive cycles with acceptable efficiency. Hence, it can be concluded that the MNPs and SMNPs are highly capable of treating contaminated waters. According to Table 6, HCl, compared to the all applied desorbent solutions , have highest effect on desorption of HA in all cycles. The results indicated that HCl solution desorbs HA in the first cycle from MNPs and SMNPs surfaces by 96.4 and 92.3%, respectively. In addition, it was seen that increasing the desorption cycles does not significantly alter the desorption efficiencies. As shown in Table 6, more than 80% of adsorbed HA could be recovered from the surface of the adsorbents in the next four cycles. High efficiency regarding HCl solution would be derived from the surface deprotonation of the adsorbent. However, it should be noted that excessive amounts of hydrogen ions might decrease the adsorption capacity of the adsorbents [46]. Hence, in this case, we washed the adsorbent using DI-water after desorption in HCl solution. Based on the results, the MNPs and SMNPs possess high potential in terms of reusability and also can be potentially regenerated through going under an acid treatment.

**Table 6:** HA adsorption and desorption percentages in 5 consecutive cycles for MNPs and SMNPs adsorbents

Cycle times	% Adsorption		% Desorption (HCl)		% Desorption (Methanol)		% Desorption (DI-water)	
	MNPs	SMNPs	MNPs	SMNPs	MNPs	SMNPs	MNPs	SMNPs
1	71.78	98.85	96.4	92.3	42.4	40.7	14.36	15.49
2	68.22	96.04	90.7	88.6	38.32	37.2	10.4	11.05
3	67.72	91.6	87.11	90.38	26.7	35.08	8.29	7.6
4	65.4	93.74	89.6	83.45	25.57	28	7.65	6.4
5	64.3	89.96	81.45	80.9	25.6	23.34	7.22	4.8

In addition, the adsorbents stability regarding the dissolved iron residues in the solution was studied. Findings revealed that in all the studied cycles for both adsorbents the dissolved iron concentrations in the solution was <0.2 mg/L, which did not exceed its maximum amount in potable water (0.3 mg/L) determined by WHO [47]. From an economic point of view, by using discussed method in this study, the operational costs can be significantly lowered; since these adsorbents have high operational reusability. Overall, our finding indicates that the MNPs and SMNPs have an acceptable level of stability which are potentially efficient adsorbents in removing HA from water with extremely low loss of activity even at acidic conditions.

## Conclusion

Magnetite nanoparticles (MNPs) and silica-coated magnetite nanoparticles (SMNPs) were successfully synthesized by applying in situ chemical co-precipitation method. Experimental results showed that the SMNPs composite had a higher HA removal efficiency, compared to the efficiency of naked MNPs in this regard. The adsorptive removal efficiencies HA to MNPs and SMNPs represented an increasing trend with an enhancement in the adsorbent dosage and temperature. Results of isotherm studies illustrated that Freundlich had more correlation with the HA adsorption experimental data than Langmuir and Temkin models, confirming the multilayer nature of HA adsorption on MNPs and SMNPs. Moreover, the experimental kinetic data was of best correlation with pseudo second-order model. In addition, the obtained results of thermodynamic studies indicated that HA adsorption on MNPs and SMNPs occurred spontaneously and is inherently endothermic. Loaded HA on both adsorbents could desorbed by using 0.1 M HCl solution; and also, the adsorbents could recycle and utilized for a long term. In addition, SMNPs were found to be a promising choice in adsorbing humic acid (HA) in a wide range of HA concentration from aqueous environments. Furthermore, we observed that the adsorption capacity of SMNPs, compared to MNPs, is higher. Hence, this adsorbent can be considered as a promising solution for treating contaminated water resources.

## Additional file

Additional file 1: The parameters and constants of kinetics and isotherms models of HA adsorption on MNPs and SMNPs. (DOCX 72 kb)

## Abbreviations

BET: Brunauer-Emmett-Teller
EDX: Energy dispersive Xray
HA: Humic acid
MNPs: Magnetite nanoparticles
NOM: Natural organic matter
SEM: Scanning electron microscopy
SMNPs: Silica-coated magnetite nanoparticles
VSM: Vibrating sample magnetometry
XRD: X-ray diffractometry
