# Supplementary material for: Silica-coated magnetite nanoparticles core-shell spheres (Fe3O4@SiO2) for natural organic matter removal
Source: J Environ Health Sci Eng. 2016 Nov 25;14:21. doi: 10.1186/s40201-016-0262-y (PMC5123275; doi:10.1186/s40201-016-0262-y)
Supplement: Additional file 1: — The parameters and constants of kinetics and isotherms models of HA adsorption on MNPs and SMNPs. (DOCX 72 kb) [file 40201_2016_262_MOESM1_ESM.docx]

**Additional file**

Kinetic models:

Pseudo-first order: *Ln(q_e_-q_t_)=lnq_e_-k_f_ t*

pseudo-second-order: *t/q_t_=t/q_e_+1/k_s_ q_e_^2^*

Intraparticle diffusion: *q_t_=k_i_ t^0.5^*

Where, q_t_ (mg/g) is the amount of adsorption capacity at a given time t; and, k_1_, and k_2_ are the rate constant of pseudo-first-order and pseudo-second-order sorption, respectively. h_0_ (mg/g min) stands for the initial sorption rates (h_0_=k_1, 2_.q_e_^n^); qe is the adsorbed amount at equilibrium state and n is the order of the kinetic model.

The intraparticle diffusion model was also used to fathom the adsorption process mechanism. k_id_ (mg/g min^0.5^) is the constant rate of the intraparticle diffusion; and, C_i_ (mg/g) is the constant which depicts the effects of boundary layer. If the value of C_i_ was equal to zero, it would indicate that the intra-particle diffusion model could be the controlling factor in determining the kinetics of the process.

Isotherm models

Langmuir: *C_e_/q_e_=C_e_/q_0_+1/k_L_q_0_*

Freundlich: *lnq_e_=lnk_F_+n^-1^ lnC_e_*

Temkin: *q_e_=B ln K_T_+B ln C_e_*

In the Langmuir model, *K*_L_ (L/mg) is an empirical constant which is related to either energy or enthalpy of the adsorption. The parameters *K*_F_ and *n* are constants of the Freundlich isotherm, which stand for the adsorption capacity and intensity, respectively.

For Temkin model, k_T_ is the equilibrium binding constant, corresponding to the maximum binding energy; and also, B=RT/b_T_, b_T_ (J/mol) is Temkin constant relating to the heat of adsorption. R (8.314J/mol K) and T (K) are the universal gas constant and solution temperature, respectively.

Adsorption thermodynamics:

Where, K_d_ (L/mg) is the distribution coefficient (i.e. the ratio of equilibrium concentration of HA on adsorbent (mg/g) to that in solution (mg/L)). The parameters ∆H^o^ and ∆S^o^ can be computed from respectively the intercept and slope of vant Hoff plots of lnK_d_ versus 1/T. The standard free energy (∆G^°^) can also be calculated using the following equation:

**Figure 1.**

The linear Langmuir (a_1_, a_2_ a_3_), Freundlich (b_1_, b2, b_3_) and Temkin (c_1_, c_2_, c_3_) isotherms for HA adsorption onto the MNPs and SMNPs at different adsorbent dosages.

b_1_

a_1_

b_2_
